# Nephroprotective activity of *Eysenhardtia polystachya* through antioxidant, H_2_S, and NO pathways

**DOI:** 10.1007/s00210-025-04331-4

**Published:** 2025-06-12

**Authors:** Emiliano de Jesús Parra-Espejel, Samara Jennifer Hipólito-Jiménez, Melissa Daniela Galicia González, Diana Montes Rojas, Viridiana Gisela Llera-Rojas, Josué A. Velázquez-Moyado

**Affiliations:** 1 Facultad de Química, Departamento de Farmacia, Universidad Nacional Autónoma de México, Ciudad Universitaria, Coyoacán, Mexico City, 04510 México; 2 Unidad de Servicios y Desarrollo Farmacéutico, Facultad de Química, Universidad Nacional Autónoma de México, Ciudad Universitaria, Coyoacán, Mexico City, 04510 México

**Keywords:** Chronic kidney disease, Coatline B, Matlaline, *Eysenhardtia polystachya*, Nitric oxide, Hydrogen sulfide, Nephroprotective, Inflammation, Ischemia–reperfusion

## Abstract

In Mexico, the use of infusions from *Eysenhardtia polystachya* in traditional medicine as a treatment for chronic inflammatory pathologies, such as urinary disorders, diabetes, and oxidative stress has been studied in the search for bioactive molecules with pharmacological properties. In this study, a methanolic extract was obtained from which Coatline B and Matlaline molecules were isolated and identified. Their pharmacological activity was subsequently evaluated as potential nephroprotective agents using both in vivo and in vitro models of acute kidney injury (AKI). The experimental results indicated that the Coatline B and Matlaline compounds exhibit antioxidant activity and play a role in nitric oxide (NO) and hydrogen sulfide (H_2_S) regulation pathways as mediators of inflammation in renal tissue. Consequently, their use as prophylactic agents in a model of AKI reduces renal tissue damage and prevents a severe decline in kidney function.

## Introduction

According to the National Institute of Public Health (INSP) of México, chronic non-communicable diseases represent one of the main causes of death worldwide, with chronic kidney disease (CKD) being one of the most relevant in the last 10 years (Paniagua-Sierra and Galván-Plata 2017). During 2017, the INSP reported a prevalence of 12.2% of CKD and a mortality rate of 51.4 per 100,000 inhabitants in Mexico (CENIDSP 2020), while worldwide, the prevalence of this disease is between 7 and 12%. According to statistics from the Pan American Organization of Health (PAHO), in 2019, the mortality rate associated with this kind of disease in the American continent was 15.6 deaths per 100,000 inhabitants (Romagnani et al. 2017).

According to the WHO statistics from 2022, heart diseases, diabetes, chronic lung disease, and cancer are the non-communicable diseases (NCDs) responsible for 74% of global deaths. Notably, kidney diseases are excluded from this list. However, pre-diabetes, heart disease, and peripheral vascular disease, along with other factors such as aging, use of nephrotoxic drugs (e.g., non-steroidal anti-inflammatory drugs (NSAIDs), allopurinol, angiotensin converting enzyme inhibitors (ACEI), some first-line antimicrobial and antivirals), kidney infections, nephrolithiasis, and genetic factors (Bilge et al. 2013; Francis et al. 2024) impair kidney function.

With the passage of time, the interaction between these factors may result in blood filtration reduction due to nephron’s dysfunction (altered excretion of water, electrolytes, drugs, and toxins from the body) accompanied by a low-grade chronic inflammation which increases the risk of chronic kidney disease (CKD) (Francis et al. 2024).

The diagnosis of renal failure is based on analytical parameters including hematuria, proteinuria, and the calculation of the glomerular filtration rate (GFR), which is considered the “Gold Standard” in clinical practice for kidney function evaluation. Currently, there is no cure for CKD. Pharmacological treatments primarily aim to manage the complications, such as diabetes, cardiovascular disorders, anemia, and hyperparathyroidism. The only specific treatments for CKD involve renal replacement therapy, either through peritoneal dialysis or hemodialysis, and, in the final stage, kidney transplantation. The therapy management is determined by a medical assessment of the patient’s health status, the risk of mortality, and the progression of the disease (Romagnani et al. 2017). Therefore, the discovery of new alternatives in therapy management to prevent CKD or improve the lifestyle of patients with CKD represents an opportunity for an underdeveloped research area.

Some of the defenses of the kidneys involve nitric oxide (NO) and hydrogen sulfide (H_2_S). Nitric oxide is a signaling molecule whose role in inflammatory diseases has been vastly described. Under physiological conditions, NO induces vasodilatation, prevents mast cell degranulation, and participates in apoptosis signaling. However, excessive production of NO may develop many inflammatory diseases such as Alzheimer’s disease, sclerosis, and arthritis. It also promotes ROS’s production, which increases pro-inflammatory cytokines and has a chemoattractive activity for leukocytes and neutrophils (Lyons, 1995, Sharma et al. 2007). Meanwhile, hydrogen sulfide is an endogenous gasotransmitter related to vascular endothelial cell proliferation, vascular smooth muscle contraction, and nervous system signaling. It reduces edema formation and leukocyte extravasation and induces glutathione (GSH) synthesis in mitochondria (Wang, 2012, Kimura, 2011). Elevated H_2_S levels induce pro-inflammatory cytokines. Some authors have reported its participation in central nervous system-related diseases, neurodegeneration, cardiovascular disease, viral infections, and renal diseases such as CKD (Dugbartey, 2023, Shahid and Bhatia 2024).

In Mexico, traditional herbal medicine has been used as an alternative for communities with limited access to medical care due to deficiencies in the national health system (Geck et al. 2020). More than 3000 species of plants have been identified and classified as medicinal plants used as adjuvants or as a treatment for different diseases (Geck et al. 2020), including diuretics, urolithiasis, and CKD.

*Eysenhardtia polystachya*—also known as Mexican kidneywood; “Palo azul,” Blue stick; “Palo dulce,” Sweet stick; Varaduz; Tlapahuaxpatli; Coathli; *Lignum nephriticum*—is a deciduous tree that can be found in warm areas located throughout Southeastern Arizona and further down until Oaxaca (Argueta, 1994). *E. polystachya* is characterized by a crown with multiple leaves in an alternating and elliptical arrangement in the shape of a bush and glandular trichomes that secrete aromatic resins. Some of the uses of the *E. polystachya* in traditional medicine have been reported as an herbal remedy for the treatment of urinary disorders such as urolithiasis. Many phytochemicals have been isolated, which include 7-hydroxy-2’,4’,5’-trymethoxyisoflavone; (3*S*)‐7‐hydroxy‐2’,3’,4’,5’,8‐pentamethoxyisoflavan; (3*S*)‐3’, 7‐dihydroxy‐2’, 4’, 5’, 8‐tetramethoxyisoflavan; soduartin: Coatline A and B; Matlaline; (α*R*)‐α,3,4,2’,4’‐pentahydroxydihydrochalcone; (α*R*)‐3’-C-β‐D‐xylopyranosyl‐α; 3,4,2’,4’‐pentahydroxydihydrochalcone; 3‐O‐acetyl‐11α; 12α–epoxy‐oleanan‐28,13β‐olide; (+)‐catechin and (+)‐catechin 3‐O‐β‐D‐galactopyranoside (Alvarez et al. 1998).

Furthermore, *Eysenhardtia polystachya* has been described to exhibit anti-inflammatory, antinociceptive (Pablo-Pérez et al. 2018; Garcia-Campoy et al. 2020), antidiarrheal, antidiabetic, antihyperlipidemic (Gutierrez and Baez 2014), potassium-sparing diuretic effect, and anti-urolithic activity (Perez et al. 1998), as well as low toxicity in vivo and in vitro models (Pablo-Pérez et al. 2016). Currently, there is no evidence of the nephroprotective activity of *E. polystachya* or its secondary metabolites Coatline B and Matlaline. In this study, we evaluate the prevention of kidney damage using these secondary metabolites as prophylaxis treatment before an acute ischemia–reperfusion injury induction. Based on the pathophysiological mechanism of the inflammatory response associated with tissular damage, we propose that nitric oxide (NO) and hydrogen sulfide (H_2_S) could be involved in the anti-inflammatory effect (Shirazi et al. 2019), and we will also describe their role as regulators of the antioxidant effects.

## Methodology

### Plant material

The plant material was kindly donated by Dr. José Fausto Rivero Cruz; the original sample was collected in Mexico City (latitude 19° 18′ 30″ N, longitude 99° 10′ 28″ W). The plant material was deposited in the Herbarium from the Faculty of Sciences, UNAM, and identified by M. in Sc. Ramiro Cruz Durán with the voucher 151,962.

### Organic extracts

For the preparation of the different organic extracts, 5 kg of bark of *E. polystachya* was submitted to an exhaustive maceration process at room temperature, employing solvents of increasing polarity in each phase. Then each organic extract obtained was submitted to a chemical fractionation with column chromatography. The structural identification of the organic compounds was done with nuclear magnetic resonance (NMR) ^13^C and ^1^H on a Varian Gemini device 200 MHz, positive electronic impact mass spectrometry (MS) was made with a JEOLJMS-AX505 HA Mass spectrometer 70 eV; all the data was verified and correlated with the reported in the literature (Acuña et al. 2009).

### Extraction and isolation of Coatline B

**Fig. 1:**
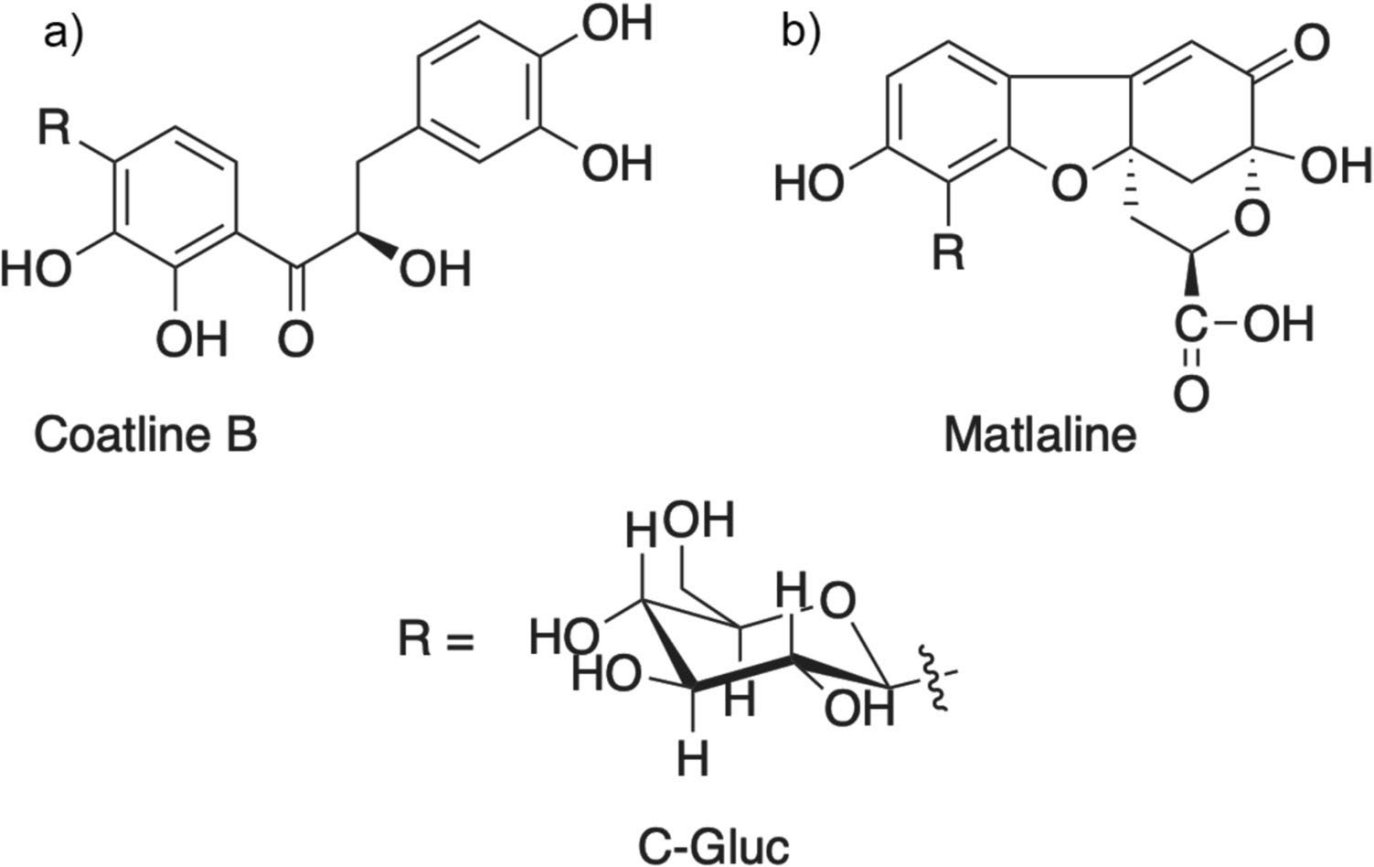
Chemical structure of Coatline B (**a**) and Matlaline (**b**) isolated from *Eysenhardtia polystachya*

The dried bark of *E. polystachya* (5 kg) was grounded into small pieces. Then the plant material was extracted through consecutive maceration three times with n-hexane (9 L), ethyl acetate (9 L), and methanol (9 L) at room temperature (22–25 °C) for 3 days. The organic extracts were concentrated *in vacuo* to dryness through a rotary evaporator. The methanolic extract had the highest yield; therefore, it was submitted to fractionation through an open column chromatography on silica gel (1 kg, Merck 70–230 mesh). The elution was made with a gradient of hexane–ethyl acetate from 10:0 to 0:10, resulting in 1284 mg of a compound with a melting point of 203–205 °C. It was then identified as Coatline B (Fig. 1A) by comparison with data from NMR and melting point, previously reported (Acuña et al. 2009; Alvarez et al. 1998).

Uncorrected melting points were measured on a Fisher-Johns apparatus. The NMR spectra were recorded on a Varian Unity Plus 400 spectrometer at 400 (^1^H) and 125 MHz (^13^C) using tetramethyl silane (TMS) as the internal standard. Chemical shifts were recorded as ∂ values.

### Coatline B

^**1**^**H NMR** (400 MHz, acetone) δ 12.93 (s, 2H), 7.94–7.81 (m, 4H), 7.74 (s, 1H), 6.74–6.62 (m, 6H), 6.55 (dd, J = 8.0, 2.1 Hz, 3H), 6.43 (d, J = 8.9 Hz, 3H), 5.15 (td, J = 7.3, 4.4 Hz, 3H), 4.88 (d, J = 9.8 Hz, 3H), 4.32 (d, J = 6.8 Hz, 5H), 4.21 (s, 1H), 4.04 (dd, J = 11.1, 5.4 Hz, 4H), 3.85 (t, J = 9.3 Hz, 3H), 3.78–3.68 (m, 3H), 3.47 (t, J = 8.8 Hz, 3H), 3.33 (dd, J = 20.5, 9.7 Hz, 6H), 2.77 (dd, J = 13.9, 7.6 Hz, 4H).^**13**^**C NMR** (101 MHz, acetone) δ 205.43, 163.8 (d, J = 27 Hz), 144.64, 143.70, 132.08, 130.52, 128.95, 120.83, 116.63, 114.92, 111.75, 110.75, 108.77, 79.13, 75.48, 73.42, 72.37, 70.55, 70.12, 48.87, 41.29, 41.01. mp. uncorrected 203—204 °C.

### Formation of matlaline

The Matlaline (Fig. 1B) was synthesized according to Acuña and collaborators’ method (Acuña et al. 2009). Briefly, we add 40 mg (6.6 mM) of Coatline B in 13.3 mL of distilled water. After that, 0.27 mL of NaOH 1 N was added and placed under constant stirring at room temperature for 4 h. Excess acetic acid (13.2 mM) was added and placed on a rotary evaporator (40 °C, 32 mBar) to eliminate the remaining solvents. The solid obtained was purified by recrystallization using the solvent-pair ethanol (EtOH) and water (H_2_O) in a proportion of 1:8 (EtOH:H_2_O). The precipitate was separated by decantation, and the excess of solvent was allowed to evaporate at room temperature.

Uncorrected melting points were measured on a Fisher Johns apparatus. The NMR spectra were recorded on a Varian Unity Plus 400 spectrometer at 400 (^1^H) and 125 MHz (^13^C) using tetramethyl silane (TMS) as the internal standard. Chemical shifts were recorded as ∂ values.

### Matlaline

^**1**^**H NMR** (400 MHz, D_2_O) δ 4.69 (s, 15H), 4.59 (s, 24H), 4.22–4.14 (m, 2H), 4.13 (s, 1H), 4.06 (s, 2H), 3.79–3.71 (m, 3H), 3.67 (s, 2H), 3.50 (hept, J = 6.4 Hz, 16H), 3.41–3.20 (m, 7H), 2.44 (s, 1H), 2.05–1.97 (m, 1H), 1.89 (d, J = 17.9 Hz, 2H), 1.81 (s, 14H), 1.04 (t, J = 6.9 Hz, 10H).^**13**^**C NMR** (101 MHz, D_2_O) δ 180.55, 130.63, 115.16, 80.60, 77.76, 74.07, 71.85, 69.30 (d, J = 21.9 Hz), 57.37, 22.69, 16.73. m.p uncorrected 221—223 °C.

### Animals

Male Wistar rats weighing between 200 and 250 g were used during all the experiments. The animals were obtained from the UNAM-UNEXA Production Center, México and were provided with free access to water and food ad libitum. Each study group consisted of at least six animals, and after each experiment, the animals were sacrificed inside a CO_2_ chamber. All experiments followed the ethical standards outlined in international guidelines for experimental pain research in animals; likewise, the guidelines set by the Official Mexican Standard for the Care and Management of Animals (NOM-062-ZOO-1999) were applied, and any effort was carried out with the purpose of minimizing pain and suffering in the animals. The number of rats used per experiment was the minimum necessary to have a reliable statistical analysis. Authorization of the protocol was obtained by the Institutional Committee for the Care and Use of Laboratory Animals (CICUAL) of the Faculty of Chemistry (CICUAL/375/19).

### Renal ischemia–reperfusion (I/R) injury

The rats were orally administered the methanolic extract obtained from *Eysenhardtia polystachya* at doses of 1, 3, 10, 30, or 100 mg/kg or the metabolites Coatline B and Matlaline at doses of 1, 3, 10, or 30 mg/kg. The P.O. administration was realized every 24 h for 5 days before the renal ischemia–reperfusion injury. A logarithmic scale was used for the doses of the extracts and secondary metabolites; the dose ranges for both the extract and the metabolites were selected based on the information reported for other metabolites (Kang et al. 2021; Kciuk et al. 2024).

After each treatment, animals were anesthetized by an intraperitoneal injection of Ketamine:Xylazine (40:10) in a single dose, maintaining anesthesia for 30 min. At the same time, the kidneys were accessed through a midline abdominal incision (approx 1.5–2 cm) (Wang et al. 2012). One of the kidneys was exposed from the retroperitoneal space so the renal pedicle was liberated. Using an absorbable surgical suture, we blocked the renal artery and vein for 15 min. While the ischemia process was happening, we exposed the contralateral side kidney, which was removed and collected as a negative control. After the kidney ischemia, we returned the kidney to the retroperitoneal space and closed the wound with nylon surgical suture. Finally, we observed the rat until its recovery (30–60 min) and monitored the animals for 2–3 days after the procedure (Cheng et al. 2022).

### Creatinine assay

Creatinine quantification is based on Jaffe’s colorimetric assay. According to the protocol of the SPINREACT® CREATININE-J Ref. 1,001,113 commercial kit: 1 mL of Picric Acid solution (17.5 mmol/L) was prepared with NaOH (0.29 mmol/L) in a 1:1 ratio. One hundred microliters of the sample (urine or serum) was added, mixed, and read at 490 nm twice: 30 s and 90 s after adding the sample to the reagent mixture.

### Urea-B assay and blood urea nitrogen (BUN) determination

Urea-B was determined using Berthelot’s colorimetric enzymatic assay. Based on the protocol of the SPINREACT® UREA-B Ref. 1,001,329 commercial kit, 10 µL of the sample (urine or serum) was added to 1 mL of buffer. The mixture was incubated at room temperature (15–25 °C) and protected from light for 10 min. After that, 1 mL of the reagent was added to a 30,000 U/L Urease-NaClO solution, incubated at room temperature and protected from light for another 10 min. At the end of the incubation time, the absorbance was read at 580 nm.

The BUN parameter was calculated using the equation proposed by Kaplan et al. (Kaplan, 1969): $\text{Blood urea nitrogen }\left(\text{BUN}\right)=\text{mg}/\text{dL Urea} \times 0.466$

### Estimated glomerular filtration rate (eGFR) calculation

Many urine parameters can be determined to estimate kidney function, like electrolytes (sodium, potassium, calcium), proteins, creatinine, urea, pH, blood urea nitrogen (BUN), glucose, and blood. In clinical practice, CKD must be diagnosed, classified, and staged by GFR (Chen et al. 2019).

Creatinine and urea were chosen as the principal biomarkers because those parameters, with the average weight (250 g) of the animals used, are used in the equation proposed by Besseling et al. (2021) (Table 1) to estimate the GFR. Additionally, urea determination was used to calculate the BUN parameter.

**Table 1:** Plasma creatinine and urea-based equation proposed by Besseling et al. (2021)

Plasma creatinine	Equation
< 52 µmol/L	$eGFR=880\times {W}^{0.695}\times {C}^{-0.660}\times {U}^{-0.391}$
≥ 52 µmol/L	$eGFR=5862\times {W}^{0.695}\times {C}^{-1.150}\times {U}^{-0.391}$

*eGFR*; estimated glomerular filtration rate, *W*; weight of the animal, *C*; serum creatinine concentration in µmol/L, *U*Urea-B; concentration in µmol/L

### Homogenized tissues

The homogenized tissues were prepared with each kidney sample (negative control, positive control (I/R damage), and each treatment of MeOH extract, Coatline B, and Matlaline) using 100 mg of tissue in 1 mL of phosphate-buffered saline (PBS). Then, they were liquefied and mixed with a homogenizer equipment. The homogenates were stored at 2–5 °C until later use.

### Glutathione (GSH) assay

The homogenate suspension was centrifuged for 15 min/1850 × *g* at 4 °C. Two hundred fifty microliters of the supernatant was taken and transferred to an Eppendorf tube. Fifty microliters of Ellman’s reagent (5,5′-dithiobis-2-nitrobenzoic acid (DTNB) and of Tris buffer 1:2 v/v) was added and mixed. The mixture obtained was incubated for 5 min at room temperature protected from light. After this period, we read absorbance at 655 nm as previously described (Velázquez-Moyado et al. 2018).

### Malondialdehyde/thiobarbituric acid (MDA/TBA) assay (lipid peroxidation)

Malondialdehyde (MDA) is a product of lipid peroxidation commonly used as a marker to evaluate tissue damage. For this assay, 225 µL of the homogenate was transferred to an Eppendorf tube and added 50 µL of phosphate buffer (pH = 7.4) and 500 µL of 10% trichloroacetic acid (TCA). The mixture was centrifuged at 606 × *g* for 10 min at 4 °C. After centrifugation, 500 µL of the supernatant was transferred, and we added to it 500 µL of 0.67% thiobarbituric acid (TBA). The samples were heated in a boiling water bath for 1 h. After this period, we read absorbance at 540 nm as previously described (Draper and Hadley 1990).

### Catalase (CAT) enzymatic activity assay

To evaluate the CAT activity, we used the protocol established by Hamza and Hadwan (Hamza and Hadwan 2020). Briefly, we prepared a solution of anilinium sulfate (0.125 M), hydroquinone (0.25 M), and ammonium molybdate (0.05% w/v); then the reagents were mixed in a proportion of 2:1:3, respectively. This solution is called “working reagent.” The tissues were washed in 1.15% (w/v) KCl solution, then filtered and diluted 1:500 with PBS buffer (pH 7.4). Then 1 mL of the tissue was mixed with 6 mL of the working reagent and with 2 mL of hydrogen peroxide (10 mM). The mixture was incubated at room temperature for 10 min protected from the light, and we read absorbance at 550 nm.

The CAT activity was reported according to the equation:

$$\text{Catalase Activity of test kU}=\frac{2.303}{\text{Time }(\text{t})}\times \text{log}\frac{\text{Absorbance of the standard }(\text{S}^\circ )}{\text{Absorbance of the sample }(\text{S})}$$

### Nitric oxide (NO) and hydrogen sulfide (H_2_S) potentiometry measure

To quantify the concentration of NO and H_2_S in the homogenized tissue samples, we used a potentiometry method. Nitrite (for NO) and sulfide (for H_2_S) electrodes were immersed to read the potential difference (ΔV) in each homogenized tissue. The ΔV register was used to calculate NO and H_2_S concentrations based on a standard curve for each compound of interest. Standard curves were previously constructed using 0.1–1.0 µM NaHS and 1.0–100 µM NaNO_2_. Total sulfides were estimated using an LS-146 AGSCM micro sulfide ion electrode and for the total NO content an LIS-146 NOCM micro nitrate ion electrode (Lazar Research Laboratories, Inc., Los Angeles, CA) (Chaiyabutr et al. 2020; Lu et al. 2022).

### Histology

The kidneys (*n* = 5) were fixed in a 10% m/v NBF solution (Formaldehyde 4%, sodium phosphate monobasic 0.4%, sodium phosphate dibasic 0.65%, methanol 1.5% and distilled water) for 24 h. The tissues were decalcified in an EDTA solution (0.35 M pH 7.8) for 72 h. Then they were dehydrated in gradual concentrations of ethanol (80%, 95%, 100% v/v) by leaving overnight in a 100% EtOH solution, clarified with xylene, and infiltrated with paraffin overnight. They were embedded in paraffin cubes and sectioned at approximately 5 µm on an RMC MR3 rotating microtome.

Picrosirius red (PSR) and hematoxylin and eosin (H&E) staining techniques were chosen because PSR staining is specific for collagen; this protein participates in cicatrization and tissular fibrosis processes (Lattouf et al. 2014). And H&E staining provides a detailed view of tissular structures and helps to identify morphological changes in the tissues (Fischer et al. 2008).

For PSR and H&E staining methods, paraffin sections were deparaffinized and rehydrated. For PSR staining, they were stained with picrosirius red (1% m/v) for 30 min and dehydrated with hydrochloric acid (0.01 M).

Hematoxylin and eosin stains were stained with hematoxylin for 10 min. Subsequently, they were washed with distilled water and stained with 0.2% eosin for 2 min.

At the end of each staining, the sections were dehydrated with EtOH (70, 96, and 100%), to finally clarify with xylene for 10 min. Mounting medium (Entellan) was then added.

### Statistical analysis

All results are presented as mean ± SEM from 6 to 7 individual repetitions. One-way ANOVA followed by Tukey’s multiple comparisons test was performed using GraphPad Prism version 10.0.0 for MacOS, GraphPad Software, Boston, Massachusetts USA, www.graphpad.com. Significance was recognized when *p* < 0.05.

## Results

The obtained yields from organic extracts were 28.39 g of hexane extract: 23.62 g of dichloromethane extract, and 486.2 g of methanolic extract. According to the literature, Coatline B conversion to Matlaline (Fig. 1B) has been described as an easy process at room temperature and in weak alkaline conditions. Because structurally, Coatline B and Matlaline are two totally different species, it was decided to compare the biological activity of Coatline B versus the methanolic extract and carry out the chemical conversion to Matlaline to identify if the biological activity is modified when analyzing both secondary metabolites (Fig. 1).

To determine the nephroprotective activity of the methanolic extract of *E. polystachya*, it was administered P.O. at doses of 1, 3, 10, and 100 mg/kg, 5 days before the induction of ischemia/reperfusion damage. Coatline B and Matlaline were evaluated in the same way at doses of 1, 3, 10, and 30 mg/kg. After the in vivo assays, serum creatinine, blood urea nitrogen (BUN), and the estimated glomerular filtration rate (eGFR) parameters were calculated to evaluate kidney function. The results show that the generation of acute kidney injury due to the ischemia/reperfusion (I/R) process increased creatinine levels (1.96 ± 0.13 mg/dL), BUN (137.10 ± 18.51 mg/dL) and the decrease in glomerular filtration rate (0.26 ± 0.053 mL/min × 100 g). The evaluation of the MeOH extract revealed that from a dose of 10 mg/kg, one can observe the prevention of decrease in renal function by the improvement of the creatinine elimination from blood (1.01 ± 0.28 mg/dL), reduction of BUN levels (117.3 ± 14.22 mg/dL) and slightly increased the glomerular filtration rate (0.557 ± 0.17 mL/min × 100 g) in comparison with the I/R injured group. Subsequently, the evaluation of Coatline B and Matlaline was carried out at doses of 1, 3, 10, and 30 mg/kg. The results showed that both metabolites improved renal function in animals with renal failure. Coatline at a dose of 30 mg/kg decreased serum creatinine levels to 1.06 ± 0.52 mg/dL, BUN levels to 85.54 ± 18.19 mg/dL, and glomerular filtration rate to 1.07 ± 0.54 mg/dL. On the other hand, Matlaline at the same dose decreased serum creatinine levels to 0.64 ± 0.15 mg/dL, BUN levels to 72.35 ± 8.92 mg/dL, and improved glomerular filtration rate by values of 1.18 ± 0.36 mg/dL. These results show that both the MeOH extract and the secondary metabolites could prevent kidney damage generated by oxygen deprivation at the nephron level (Table 2).

**Table 2:** Urine parameters serum creatinine, blood urea nitrogen, and calculated eGFR as kidney function tests

	Serum creatinine (mg/dL)	Blood urea nitrogen (BUN) (mg/dL)	Estimated glomerular filtration rate (eGFR) (mL/min × 100 g)
Reference values	0.54 ± 0.02	39.02 ± 1.74	1.2 ± 0.04
Treatment			
Sham	0.59 ± 0.09	62.62 ± 12.61	1.77 ± 0.46
I/R	1.96 ± 0.13*	137.10 ± 18.51*	0.26 ± 0.053*
Coa 1	1.66 ± 0.23*	144.12 ± 21.47*	0.27 ± 0.031*
Coa 3	1.35 ± 0.25*	108.62 ± 14.59*	0.36 ± 0.068*
Coa 10	1.09 ± 0.32*^a^	93.87 ± 24.05	0.54 ± 0.16*
Coa 30	1.06 ± 0.52*^a^	85.54 ± 18.19^a^	1.07 ± 0.54*^a^
Mat 1	1.66 ± 0.19*	100.52 ± 21.26*	0.29 ± 0.05*
Mat 3	1.18 ± 0.34*^a^	86.27 ± 16.01^a^	0.54 ± 0.22*
Mat 10	0.73 ± 0.17^a^	76.35 ± 12.14^a^	0.964 ± 0.36*^a^
Mat 30	0.64 ± 0.15^a^	72.39 ± 8.92^a^	1.18 ± 0.36^a^
Ext 10	1.01 ± 0.28^a^	117.3 ± 14.22*	0.557 ± 0.17*
Ext 100	0.84 ± 0.16^a^	73.08 ± 16.72^a^	0.798 ± 0.26*^a^

Values are expressed as mean ± SD (*n* = 6). Statistical comparison was performed using one-way ANOVA followed by Tukey’s test with a *p*-value of 0.05. Statistically significant difference (*p* < 0.05) is indicated with an asterisk (*) for the Sham group and with a superscript “a” (^a^) for the I/R induced damage group

To support “Sham” experimental results, serum creatinine and BUN reference values were taken from Thammitiyagodage et al. (2020), and the estimated glomerular filtration rate (eGFR) reference value was taken fromPais et al. (2022) and Thammitiyagodage et al. (2020)

**Fig. 2:**
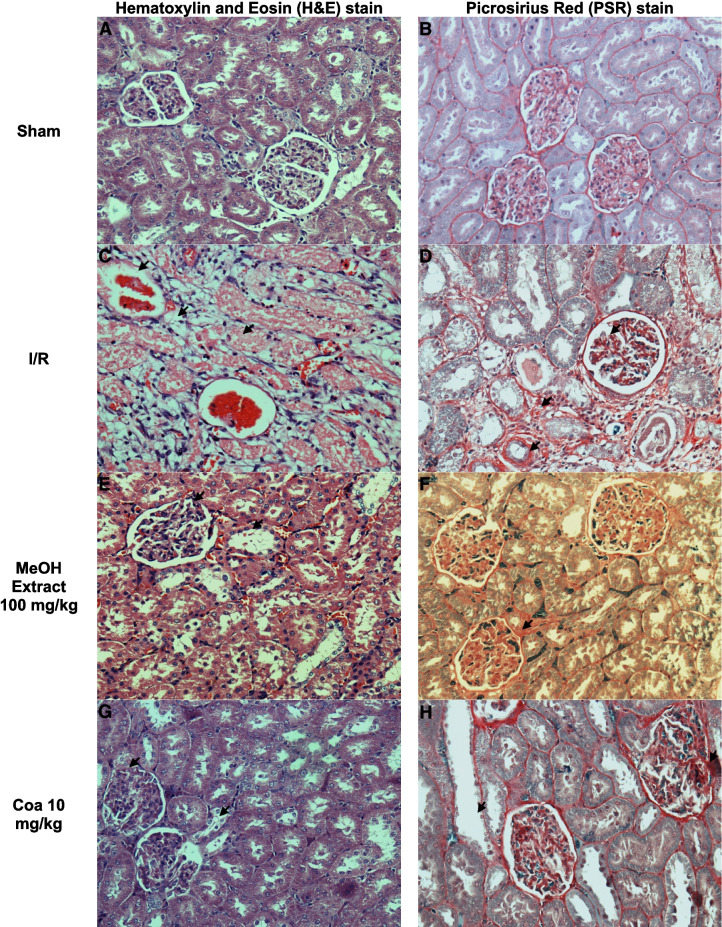

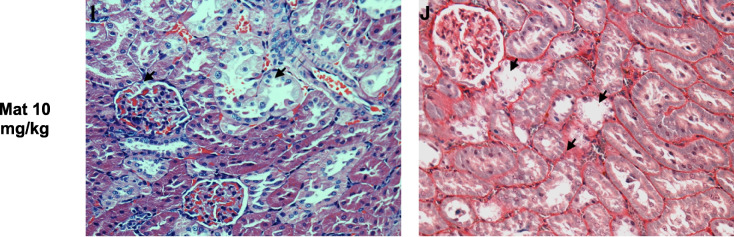
Histology of the sham group kidney tissue (**A**, **B**). Renal acute injury induced by I/R injury affected kidney tissue; the histology of the I/R group showed immune system cellular infiltration, tubular and interstitial damage, and multiple collagen networks associated with the cicatrization of the kidney tissue after I/R injury (**C**, **D**). In MeOH extract of *E. polystachya* 100 mg/kg dose treatment (**E**, **F**), Coatline B (Coa) 10 mg/kg treatment (**G**, **H**) and Matlaline (Mat) 10 mg/kg treatment (**I**, **J**) kidney samples showed a decrease in those indicators

To determine the effect of the extracts and secondary metabolites on the cellular structure, histological sections of the injured tissues were made. For this purpose, hematoxylin and eosin (H&E) and Picrosirius red (PSR) staining were made. The results show that the damage generated by the ischemia process shows thinning in the region of the glomerulus, tissue disorganization, and loss of the brush border of the tubular cells compared to Sham animals (arrows, Fig. 2A and C); PSR allows us to identify the regions where the formation of fibrotic tissue is found; these lesions are located in the region of the glomerulus with collagen accumulations (Fig. 2B and D). The animals pretreated with the MeOH extract at a dose of 10 mg/kg showed a decrease in damage at the level of the glomerulus, in addition to a reduction in the loss of the brush border, and collagen deposits were also observed in the juxtaglomerular regions (Fig. 2E and F).

The animals treated with Coatline B 10 mg/kg presented a decrease in the loss of the brush border of the epithelial cells and less accumulation of intermediate filaments in the periphery of the glomerulus (Fig. 2G and H). The animals treated with Matlaline 10 mg/kg did not present malformations at the tubular, and there is little loss of the microvilli of the nephron cells (Fig. 2I). The normal shape and integrity of the nephron capsule can be observed. In the PSR stain, it can be observed that there is less formation of fibrotic tissue; the definition of the cells within the tissue can be observed (Fig. 2J).

Once the activity of the extracts and secondary metabolites on kidney functionality was evaluated, the next point was to determine if the prevention of damage generated by the hypoxia process in the tissue was related to the decrease in oxidative stress. For this purpose, homogenized tissues were used to evaluate antioxidant activity through enzymatic assays and identification of effect biomarkers (Catalase, Glutathione, and Malondialdehyde).

**Fig. 3:**
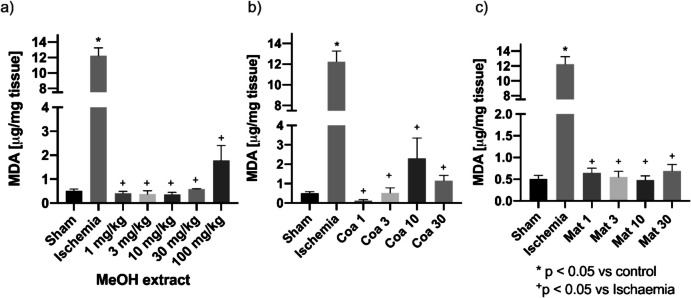
Malondialdehyde (MDA) concentration as an indicator of lipid oxidative damage in kidney tissue samples. **a** Methanolic extract of *E. polystachya*. **b** Coatline B isolated from the methanolic extract of *E. polystachya*. **c** Matlaline obtained from the hydrolysis reaction of Coatline B. Data are presented as mean ± SEM. Statistical comparison was performed using one-way ANOVA followed by a *post-hoc* Tukey’s test

The TBARS (thiobarbituric acid reactive substances) assay was used to quantify MDA as a biomarker of the effect of oxidant stress to determine hypoxia damage on structural molecules such as membrane phospholipids. The results obtained from the in vitro tests using homogenized tissues showed that the methanolic extract prevents the oxidative damage generated by the ischemia process. Interestingly, it can be observed that at a dose of 100 mg/kg the extract begins to lose effectiveness (Fig. 3A), which suggests its activity as a prooxidant in high doses. On the other hand, the secondary metabolites—Coatline B and Matlaline—prevented the lipoperoxidation process in the different doses evaluated (Fig. 3B and C) in comparison with the positive control group (I/R damage).

**Fig. 4:**
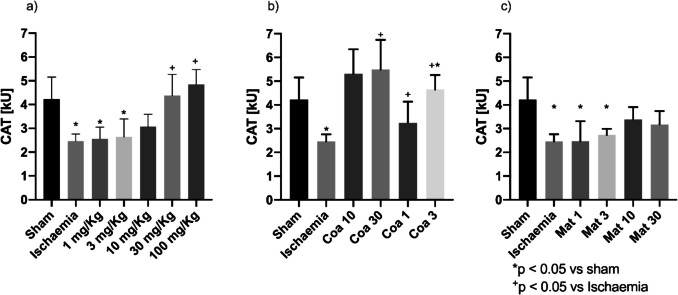
Catalase (CAT) activity as an indicator of antioxidant activity in kidney tissue samples. **a** Methanolic extract of *E. polystachya*. **b** Coatline B isolated from the methanolic extract of *E. polystachya*. **c** Matlaline obtained from the hydrolysis reaction of Coatline B. Data are presented as mean ± SEM. Statistical comparison was performed using one-way ANOVA followed by a *post-hoc* Tukey’s test

**Fig. 5:**
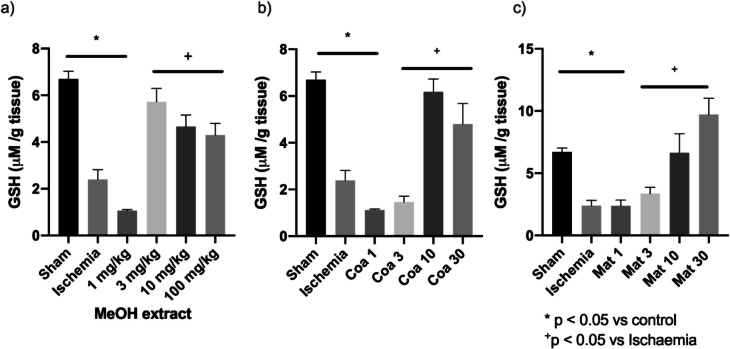
Reduced glutathione (GSH) concentration as an indicator of antioxidant activity in kidney tissue samples. **a** Methanolic extract of *E. polystachya*. **b** Coatline B isolated from the methanolic extract of *E. polystachya*. **c** Matlaline obtained from the hydrolysis reaction of Coatline B. Data are presented as mean ± SEM. Statistical comparison was performed using one-way ANOVA followed by a *post-hoc* Tukey’s test

To determine the antioxidant activity, two of the most important systems were evaluated: reduced glutathione as a non-enzymatic antioxidant and catalase as an enzymatic antioxidant. The catalase enzymatic assay showed that the activity of this enzyme increases in a dose-dependent manner respecting the positive control at doses of 3, 10, and 30 mg/kg of Coatline B (Fig. 4B). In those groups of Coatline B, catalase activity reaches similar levels in comparison with the sham group. In contrast to the doses of 1 mg/kg and 3 mg/kg of Matlaline did not prevent the loss of function derived from kidney injury (Fig. 4C); rather, the results show an upward trend in the prevention of damage at higher doses; however, more results are needed to corroborate this theory. On the other hand, the reduced glutathione assay showed that the concentration of this antioxidant peptide decreases after an I/R injury; yet, in the groups treated with the methanolic extract at doses of 3, 10, and 100 mg/kg, the concentration is not showing a significant difference respecting healthy individuals (Fig. 5A). Similarly, it is observed that the protection generated by secondary metabolites starts to occur from a dose of 10 mg/kg (Fig. 5B and C).

**Fig. 6:**
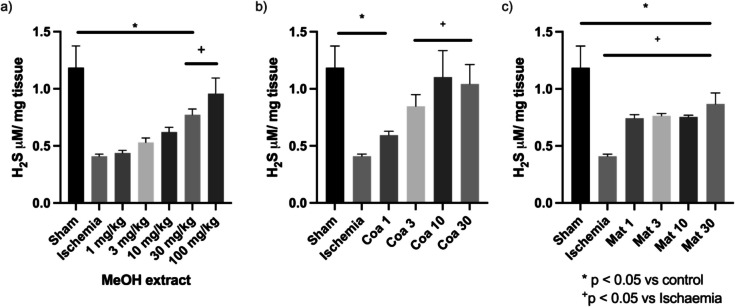
Hydrogen sulfide (H₂S) concentration in kidney tissue samples. **a** Methanolic extract of *E. polystachya*. **b** Coatline B isolated from the methanolic extract of *E. polystachya*. **c** Matlaline obtained from the hydrolysis reaction of Coatline B. Data are presented as mean ± SEM. Statistical comparison was performed using one-way ANOVA followed by a *post-hoc* Tukey’s test

Finally, the concentration of two gasotransmitters—H_2_S and NO—which participate in the regulation of inflammatory processes and which are related to the development of physiological alterations such as renal failure, will be determined. Ischemia damage depletes H_2_S levels, as has been previously reported in acute kidney injury models (Pieretti et al. 2020). The results obtained showed that the methanolic extract prevents the decrease in the H_2_S concentration at doses of 30 and 100 mg/kg. It is observed that the dose of 100 mg/kg maintained the levels of this gasotransmitter in similar quantities to the ones in healthy animals (Fig. 6A). Similarly, the results of Coatline B reveal that there is a prevention of the decrease in H_2_S levels in animals with ischemic damage at doses of 3, 10, and 30 mg/kg of Coatline B (Fig. 6B). Finally, there is no dose–response relationship on the regulation of H_2_S in the different doses of Matlaline that were evaluated; yet, it is observed that there is a prevention of depletion of this gasotransmitter for all doses. Therefore, it can be said that the extract and the isolated metabolites participate in preventing the decrease in the concentration of H_2_S.

**Fig. 7:**
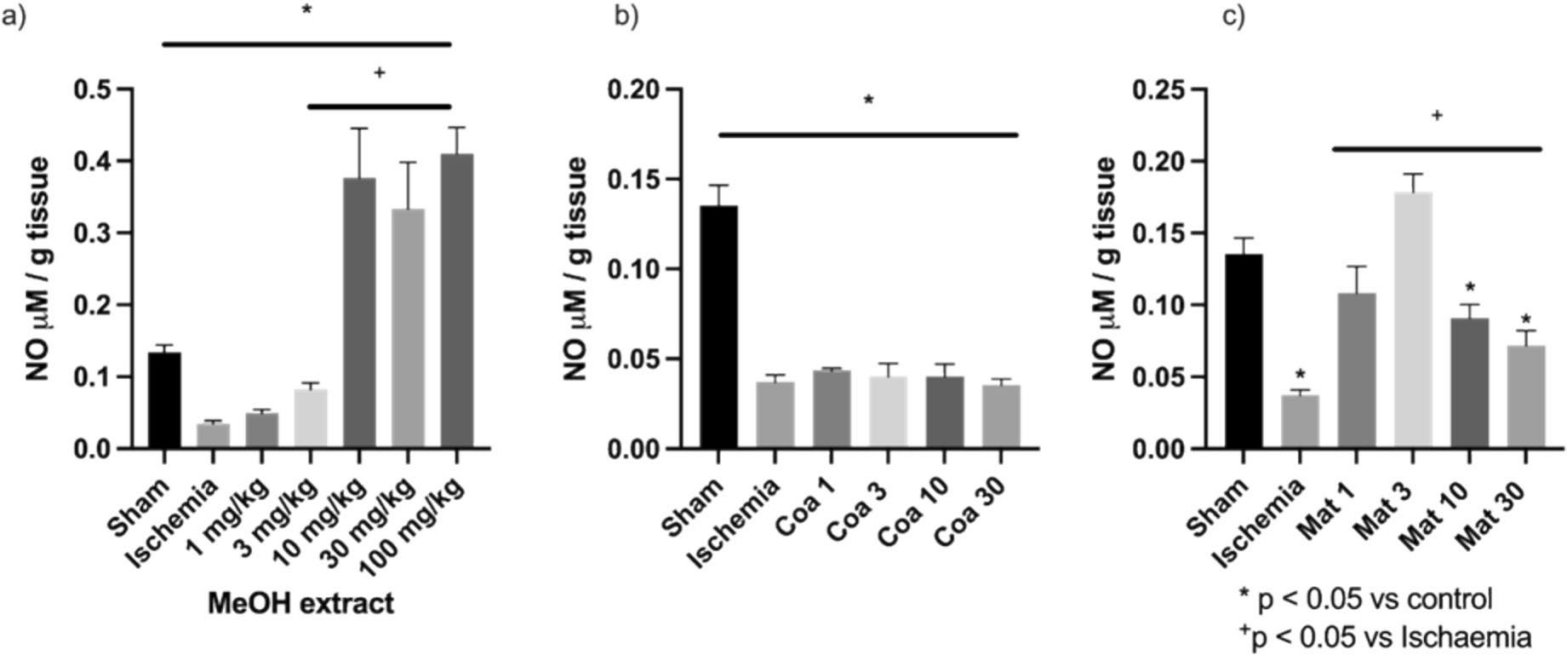
Nitrite concentration as an indicator of nitric oxide (NO) production in kidney tissue samples. **a** Methanolic extract of *E. polystachya*. **b**) Coatline B isolated from the methanolic extract of *E. polystachya*. **c** Matlaline obtained from the hydrolysis reaction of Coatline B. Data are presented as mean ± SEM. Statistical comparison was performed using one-way ANOVA followed by a *post-hoc* Tukey’s test

At the same time, the amount of nitric oxide contained in the injured kidneys was evaluated. The results of the quantification of NO showed that the animals with ischemic injury presented a significant decrease in levels of this gasotransmitter when compared to the Sham animals. The evaluation of the methanolic extract revealed that the nephroprotective effect was observed at doses of 10, 30, and 100 mg/kg. In addition, it was observed that there is a significant increment in the concentration of this second messenger (Fig. 7A). Interestingly, it is observed that Coatline B does not modify the production of NO at the different doses evaluated, since the levels are comparable to that of animals without treatment (Fig. 7B). In the case of the isolated metabolites, the administration of Coatline B does not modify NO concentrations when ischemic damage is induced. In the case of Matlaline, the results showed the maintenance of NO levels from the dose of 1 mg/kg, until increasing these levels to the dose of 3 mg/kg; however, interestingly, it can be observed that the increase in the dose generates a tendency to decrease the values of this mediator at doses of 10 and 30 mg/kg (Fig. 7C).

## Discussion

CKD is characterized by local and systemic inflammation. This physiological condition stimulates pro-inflammatory cytokines production, increases levels of oxidative stress and creatinine in blood, reduces GFR, and causes metabolic waste retention, cardiovascular conditions, and imbalance in the body fluids (Levey et al. 2015). Some authors have proposed murine models of renal ischemia/reperfusion (I/R) injury for acute kidney injury (AKI) and CKD because both pathologies affect renal function, and the consequences of each one are similar (Harwood et al. 2022). Renal I/R injury produces inflammation and oxidative stress in the kidneys; these factors contribute to generating renal tissue damage, activated immune system cells (like neutrophils and macrophages), tubular damage, necrosis, apoptosis, and pyroptosis (Baer et al. 2020; Piko et al. 2023; Kurts et al. 2013).

Currently, there is no treatment available that can prevent or reduce kidney damage at various stages. The existing treatments mainly focus on managing different comorbidities such as diabetes, obesity, hypertension, and chronic medication use. However, over the long term, these conditions continue to impair kidney functionality, ultimately leading to kidney failure. Natural products are viewed as an inexhaustible source of new compounds with therapeutic potential. Numerous reports highlight various phytocompounds and plant extracts that exhibit cytoprotective activity against kidney damage in ischemia–reperfusion processes. It has been found that the activation and prolongation of the inflammatory immune response are primarily influenced by reducing levels of pro-inflammatory cytokines, achieved by blocking, among other pathways, the nuclear transcription factor. In addition, there is an increase in anti-inflammatory cytokines and other molecules like adenosine, which also exert an anti-inflammatory effect (Aboutaleb et al. 2019; He et al. 2017; El Morsy et al. 2015). Some plants reported as nephroprotective agents include *Crateva nurvala*, which modifies levels of TNF-α and IL-6 (Choucry et al. 2018), and *Crocus sativus* L., which also alters the levels of TNF-α and ICAM (Mahmoudzadeh et al. 2017). *Rosa laevigata* Michx affects levels of NF-κB, IL-6, and IL-1β (Zhao et al. 2016). Additionally, there are reports on the activity of secondary metabolites, including Cannabidiol, Cryptotanshinone, Oleanolic Acid, Curcumin, Crocin, Vanillin, and Dioscin, that act as regulators of the inflammatory cascade through the modulation of TNF-α, IL-1β, IL-12, IL-18, IFN-γ, and TGF-β (Fouad and Al-Melhim 2017; Abou-Hany et al. 2018; Gamede et al. 2019; Bai et al. 2019). The use of *E. polystachya* infusions in traditional medicine for various urinary disorders has documented reports on its antidiabetic, anti-inflammatory, antiparasitic, and bactericidal properties. However, this is the first study to describe its nephroprotective activity through the organic extract and two main metabolites of the plant, Coatline B and Matlaline (Garcia-Campoy et al. 2020; Garcia Campoy et al. 2018; Pablo-Pérez et al. 2018).

The results showed that Coatline B, Matlaline, and the MeOH extract reduced BUN and serum creatinine levels compared to the I/R group (Table 2). To our knowledge, these are the first results demonstrating their activity as nephroprotective agents. These findings associate the administration of *E. polystachya* extract and its metabolites as a prophylactic therapy with the reduction of renal damage caused by I/R. An improvement in the glomerular filtration rate was also observed, suggesting the maintenance of renal function. The results are aligned with the structural findings, where maintenance of renal structures is observed with the treatments of the methanolic extract and the administration of secondary metabolites. Interestingly, it can be observed that Matlaline has better activity in improving kidney function, both at a functional and structural level, preventing the formation of fibrotic tissue, infiltration of inflammatory cells in the renal tubules, tubular dilation, glomerular atrophy, loss of podocytes, and microvilli (Fig. 2).

The concept of “oxidative stress” has been used since 1985 (Sies, 1985) to describe the imbalance between reactive species (oxidant species) and antioxidants. The principal reactive species produced by metabolism are the reactive oxygen species (ROS), reactive nitrogen species (RNS), and lipid peroxyl radicals. These species can react with several biomolecules in the organism and, in consequence, generate DNA damage, protein alterations, membrane damage, cellular senescence, apoptosis, among many others (Libby et al. 2002; Kelley et al. 2019).

In the context of I/R damage, oxidative stress promotes inflammation and a pro-oxidant microenvironment. This environment promotes the production of MDA, the final product of lipid peroxidation, which primarily occurs on polyunsaturated fatty acids (Esterbauer et al. 1990). When MDA levels increase, the metabolic pathways that normally inactivate this electrophilic product are saturated; this phenomenon allows MDA to bind to nucleophilic molecules such as proteins and nucleic acids, causing cell damage and death (Nam, 2011). Additionally, MDA quantification has been widely used as a biomarker of effect for oxidative stress damage in I/R lesions, which shows a correlation between increased MDA levels and the loss of organ function (Iłżecki et al. 2021). The experimental findings from this study demonstrated that the administration of the methanolic extract and the metabolites isolated from *E. polystachya* reduce MDA levels (Fig. 3), and, in consequence, decrease lipid peroxidation associated with the oxidant stress characteristic of I/R damage. These results are important because MDA is recognized as the most important mutagenic agent produced by lipid peroxidation. Furthermore, it is also known that one of the main systems responsible for the reduction of hydroperoxides is the glutathione peroxidase system, which uses GSH as a reducing agent (de Haan et al. 2005).

It was proposed to evaluate antioxidant mechanisms to determine the influence of the methanolic extract, Coatline B, and Matlaline in preventing the induction of oxidative stress. During the I/R procedure, hypoxia promotes the release of ROS, which act as messengers of tissue damage and activate pathways related to the loss of membrane structure, damage to the mitochondrial respiratory chain, alterations in calcium-dependent signaling pathways, and cell death. Some authors (Lorente et al. 2016) have described the joint participation of enzymatic and non-enzymatic systems that regulate reactive oxygen species (ROS) production.

To evaluate the enzymatic antioxidant systems, we chose to evaluate catalase enzyme activity, which is responsible for reducing peroxides, specifically hydrogen peroxide (H_2_O_2_). This molecule is one of the main products of other enzymatic antioxidant systems such as XO and SOD, and it is also a substrate for chemical reactions that catalyze the formation of other reactive species, such as peroxyl radical (•OH). The reaction speed of •OH increases the probability of it reacting with endogenous biomolecules, damaging organelle structures and interfering with renal filtration (Ho and Shirakawa 2022). High doses of methanolic extract, 30 and 100 mg/kg, showed an increment of catalase activity (Fig. 4A). The administration of 3, 10, and 30 mg/kg Coatline B doses showed an increment of catalase activity in comparison with the I/R group (Fig. 4B). The effects that were observed could be associated with a mayor availability of free catalase in renal tissues. On the other hand, the non-enzymatic antioxidant system evaluated was the concentration of GSH in the tissues, due to its role in inactivating ROS and other electrophilic molecules capable of reacting with endogenous molecules that could cause damage to the cell structures and function. The results showed that the methanolic extract of *E. polystachya* prevented the decrease in GSH concentration when it was administered before subjecting the individuals to the I/R process (Fig. 5A). When we evaluated the isolated metabolites, the results showed that both Coatline B and Matlaline prevent the decrease in GSH concentration caused by I/R (Fig. 5B and C). These observations may be related to the structural arrangement of the molecule, as it allows them to act as proton donors, participating in the reduction of ROS in a manner analogous to what has been reported with other molecules with a structure similar to chalcones (Lahsasni et al. 2014) and flavonoids whose antioxidant activity has already been well documented (Zahra et al. 2017; Galisteo et al. 2004; Zhou et al. 2017). Both enzymatic and non-enzymatic antioxidant assays showed that Coatline B and Matlaline show antioxidant activity, which may be related to the chemical structure of these secondary metabolites. In addition, the methanolic extract demonstrates antioxidant activity likely due to the presence of both secondary metabolites and other unidentified molecules that could also have antioxidant effects.

Finally, the anti-inflammatory activity of the methanolic extract—Coatline B and Matlaline—was evaluated by quantifying NO and H_2_S, which are two of the most relevant gasotransmitters in the signaling of inflammatory processes (Xie et al. 2016; Liu et al. 2014; Arranz et al. 2003). The methanolic extract showed that it prevents the decrement of H_2_S when it is administered at doses of 10 and 100 mg/kg (Fig. 6A); meanwhile, the prevention of the decrement of NO required doses upper 3 mg/kg. However, the doses of 10 to 100 mg/kg show an elevation in NO concentrations (Fig. 7A). These findings show that the methanolic extract has a dose–response anti-inflammatory effect acting in both molecules, NO and H_2_S, signalization pathways.

When we evaluated the metabolites isolated from *E. polystachya*, Coatline B at doses of 3, 10, and 30 mg/kg prevented the decrease in H_2_S concentrations (Fig. 6B). However, for NO, no significant difference was observed between the concentrations in the positive control group and the groups treated with Coatline B (Fig. 7B). In contrast, Matlaline prevented the decrease in H_2_S concentration associated with I/R damage but did not reach the values of healthy animals (Fig. 6C). Additionally, Matlaline at doses of 1 and 3 mg/kg prevented the decrease in NO concentration when it is administered as prophylaxis treatment before the induction of I/R damage, showing values similar to those obtained from the samples of healthy individuals (Fig. 7C). This could be explained by the fact that Coatline B may promote H_2_S synthesis by inducing the production of this gasotransmitter through the activation of enzymes cystathionine β-synthase (CBS) and cystathionine γ-lyase (CSE) (El-Sayed et al. 2021; Younis et al. 2016; Zuhra et al. 2022). In contrast, the activity of Matlaline may be more associated with the induction of NO synthesis through the activation of the enzyme NOS present in the epithelial tissue of the kidneys (Duarte et al. 2014; Haenen and Bast 1999). Those insights are supported by the results of the methanolic extract (Figs. 6A and 7A), which show a prevention of the decrease in NO and H_2_S when both metabolites and other molecules are involved. However, a NO synthesis above normal values was observed at the highest doses of the methanolic extract. This could be associated with pathological activity that may be causing tissue damage due to the induction of pro-inflammatory pathways, favoring immune system cell activation and generating an inflammatory microenvironment probably through the TLR4/MyD88/NF-κB/iNOS pathway (Jin et al. 2019; Ma et al. 2022). Further tests are required to verify and specify the mechanism of action that these metabolites may have on the activation of these inflammatory/anti-inflammatory pathways.

In summary, we observed that methanolic extract, Coatline B, and Matlaline can decrease inflammation levels by acting on the NO and H_2_S pathways. Additionally, the molecular structures of Coatline B and Matlaline enable them to act as antioxidants, and in consequence, preventing the tissular damage caused by oxidative stress. As a result of the combination of these factors, we can affirm that the extract and the metabolites we evaluated could reduce the kidney damage caused by chronic inflammation in CKD.

## Conclusions

Coatline B and Matlaline (secondary metabolites of *E. polystachya*) demonstrated anti-inflammatory effects, antioxidant activity, and reduced oxidative stress-induced tissue damage. Coatline B and Matlaline likely have a combined mechanism of action, which includes the activation of NO and H_2_S pathways as well as the promotion of both non-enzymatic and enzymatic antioxidants. In conclusion, Coatline B and Matlaline are promising natural prophylaxis therapies of CKD and other inflammatory kidney diseases. Nowadays, natural remedies are still an alternative source of healthcare strategies for treating many chronic diseases and represent an important source of bioactive molecules, with potential therapeutic properties.

## Data Availability

All source data for this work (or generated in this study) are available upon reasonable request.

## Abbreviations

ACEI: Angiotensin converting enzyme inhibitors
AKI: Acute kidney injury
ANOVA: Analysis of variance
CAT: Catalase
CBS: Cystathionine β-synthase
CICUAL: Institutional Committee for the Care and Use of Laboratory Animals
CKD: Chronic kidney disease
Coa: Coatline
CSE: Cystathionine γ-lyase
DNA: Deoxyribonucleic acid
DTNB: 5,5'-Dithiobis-2-nitrobenzoic acid
EDTA: Ethylenediaminetetraacetic acid
Ext: Extract
eGFR: Estimated glomerular filtration rate
EtOH: Ethanol
GSH: Glutathione
GFR: Glomerular filtration rate
H&E: Hematoxylin and eosin
H_2_O: Water
H_2_S: Hydrogen sulfide
IL: Interleukine
INSP: National Institute of Public Health
I/R: Ischemia-reperfusion
KCl: Potassium chloride
Mat: Matlaline
MDA: Malondialdehyde
MeOH: Methanol
mM: Millimolar
mBar: Millibar
MS: Mass spectrometry
NaClO: Sodium hypochlorite
NaHS: Sodium hydrosulfide
NaNO_2_: Sodium nitrite
NaOH: Sodium hydroxide
NMR: Nuclear magnetic resonance
NO: Nitric oxide
NOS: Nitric oxide synthase
NSAIDs: Non-steroidal anti-inflammatory drugs
PBS: Phosphate-buffered saline
P.O.: Oral administration
PSR: Picrosirius red
ROS: Reactive oxygen species
SOD: Superoxide dismutase
TCA: Trichloroacetic acid
TBA: Thiobarbituric acid
TBARS: Thiobarbituric acid reactive substances
TMS: Tetramethyl silane
TNF: Tumor necrosis factor
UNAM: National Autonomous University of Mexico
UNEXA: Animal Experimentation Unit
XO: Xanthine oxidase
